# Comparison of Clinical Outcomes, Visual Quality and Visual Function of Two Presbyopia-Correcting Intraocular Lenses Made from the Same Material, but with Different Design and Optics

**DOI:** 10.3390/jcm10153268

**Published:** 2021-07-24

**Authors:** Ladislav Viktor Nováček, Marie Němcová, Kateřina Tyx, Kristýna Lahodová, Leoš Rejmont, Pavel Rozsíval, Pavel Studený

**Affiliations:** 1 Department of Ophthalmology, Institute of Aviation Medicine Prague, 160 00 Prague, Czech Republic; nemcova@ulz.cz (M.N.); tyx@ulz.cz (K.T.); lahodova@ulz.cz (K.L.); pavel.rozsival@fnhk.cz (P.R.); 2 Department of Ophthalmology, 1st Faculty of Medicine, Charles University and the Military University Hospital Prague, 121 08 Prague, Czech Republic; leos.rejmont@lf1.cuni.cz (L.R.); pavel.studeny@fnkv.cz (P.S.); 3 Department of Ophthalmology Charles University Prague, School of Medicine Hradec Králové, 500 03 Hradec Králové, Czech Republic

**Keywords:** presbyopia, multifocal intraocular lenses (MIOL), cataract, visual outcomes, visual function, contrast sensitivity, dysphotopsia, posterior capsule opacification, 677MY, 839M

## Abstract

This semi-prospective, parallel, comparative investigation evaluated the clinical outcomes and quality of vision (contrast sensitivity, visual function, dysphotopsia, spectacle use, overall satisfaction) after mono- or bilateral implantation of two presbyopia-correcting intraocular lenses (IOL)—the Liberty^®^ 677MY or the AT LISA^®^ tri 839M—in 50 eyes of 25 cataract patients. Clinical outcomes were assessed 3 and 12 months postoperatively. Eighty-nine percent of eyes implanted with the Liberty IOL and 59% of eyes implanted with the AT LISA IOL achieved a refractive outcome ±0.5 diopters of the target (emmetropia). Refractive outcomes were stable with both lenses. The proportions of eyes with 20/20 uncorrected distance visual acuity (UDVA) and 20/20 uncorrected near visual acuity (UNVA) were higher in the Liberty group than in the AT LISA group (UDVA: 56% vs. 41%; UNVA: 83% vs. 66%). Optical quality assessment results were comparable for the two IOLs. Superior photopic contrast sensitivity was found with the Liberty lens. The rate of Nd:YAG capsulotomy at the 12-month follow-up was 16.7% in the Liberty group and 40.6% for the AT LISA IOL. Considering that both lenses are made from the same material, we propose that the noted differences in clinical outcomes may derive from differences in design and optical surface between the two IOLs.

## 1. Introduction

A growing reliance on intermediate vision in the modern world has contributed to an evolution in presbyopia-correcting intraocular lenses and the introduction of new trifocal intraocular lens (IOL) technologies that can provide functional vision without correction across a full range of distances. Although the latest technologies have brought an extended depth of focus and light-adjustable intraocular lenses, a recent survey performed by the European Society of Cataract and Refractive Surgery (ESCRS), based on the responses of 2149 doctors, has shown that the largest percentage (63%) of surgeons still prefer trifocal intraocular lenses when choosing an IOL for presbyopia correction [1]. Similar surgeons’ preferences were reported by Logothetis et al. in a recent paper [2].

These IOLs have become the focus of recent clinical research aiming to reveal possible differences between trifocal IOL models, highlighting the advantages and possible target group of each type. In addition to the refractive outcome and visual acuity at multiple distances, measures of visual quality are of particular interest. Dysphotopic events and compromised vision in sub-optimal light conditions are reported relatively frequently with multifocal IOLs, especially those with diffractive optical surface technologies [3,4]. Cataract surgeons cite problems with the nighttime quality of vision and loss of contrast visual acuity as their most common concerns relating to presbyopia-correcting IOLs [1].

Visual outcomes after cataract surgery and the long-term stability of the results are influenced by multiple features of an IOL [5,6,7,8,9]. Both IOL material and optical design play major roles in the final visual outcome [10,11,12,13]. In addition, IOL material, optic edge profile, and haptic design influence the development of posterior capsule opacification (PCO), which usually results in decreased vision [14,15,16]. PCO is easy to treat by performing Nd:YAG laser capsulotomy, but the procedure can lead to IOL damage, intraocular pressure (IOP) elevation, cystoid macular edema and retinal detachment [17].

Our study aimed to compare the refractive outcomes, visual performance, and quality of vision (contrast sensitivity, optical quality, visual function, dysphotopsia, spectacle use and overall satisfaction) after cataract surgery with bilateral implantation of two different trifocal IOLs that are made from the same material, but differ in their optical surface characteristics and other design features.

## 2. Materials and Methods

The current investigation was carried out at a single center, the Department of Ophthalmology, Institute of Aviation Medicine, Prague, Czech Republic. The study was conducted according to the guidelines of the Declaration of Helsinki [18] and approved by the Ethics Committee of the University Hospital Hradec Králové (Approval number: 20210 J01; date of approval: 25 May 2021). Prior to any intervention, each patient was given detailed information about the surgery and the objectives of our study. Each subject gave his or her written consent about contributing to and participating in this research.

All patients had bilateral age-related cataracts amenable to surgery using the phacoemulsification method, with implantation of a capsular bag IOL. All patients had normal ocular conditions, including normal anterior segment, healthy retinal status, and clear intraocular media apart from the cataract.

Special attention was paid to not including any patients with a history of intraocular or corneal surgery, congenital eye abnormality, or eye trauma. Patients with non-age-related cataracts, irregular astigmatism, diabetic retinopathy, age-related macular degeneration or any other retinal diseases, glaucoma, amblyopia, uveitis, pseudoexfoliation syndrome, iris neovascularization, severe myopia, or abnormal pupillary function were excluded.

All surgeries were performed by the same surgeon (L.N.) between April 2017 and September 2019 using the phacoemulsification technique. After local anesthesia, a clear corneal incision was made at 135° with a length of 1.7 mm that was later extended to 2.2 mm for IOL implantation. A paracentesis was made at 45°. After filling up the anterior chamber with the viscoelastic material, and removing the cataractous lens, the AT LISA tri 839MP IOL (Carl Zeiss Meditec AG, Jena, Germany) or Liberty 677PMY IOL (Medicontur Medical Engineering Ltd., Zsámbék, Hungary) was implanted. IOL selection for each patient was affected by financial issues. The same IOL was implanted bilaterally.

The AT LISA tri 839MP and Liberty 677PMY IOLs are made of the same hydrophilic–hydrophobic acrylic copolymer material (Benz-25 (Benz Research & Development, Sarasota, FL, USA)), but they have different designs. The AT LISA tri 839MP has an overall diameter of 11 mm, an optic diameter of 6.00 mm, and plate haptics. The Liberty 677PMY is larger in terms of overall diameter and has a double-loop haptic design. The optic of both IOLs is based on diffractive optical principles, but they use different approaches to achieve trifocality. Further technical features of the two study devices are summarized in Supplementary Table S1. The Liberty IOL was implanted using either the Medjet B1B or the Medjet MA 2.2 injector by Medicontur. The AT LISA IOLs come preloaded in the 2BLUEMIXS^®^ 180 injector-cartridge set. After complete removal of viscoelastic material, the incisions were left sutureless in all cases. Antibiotic and antimicrobial eye drops were administered intraoperatively in each case to reduce the risk of surgical infections, and antibiotic and anti-inflammatory eye drops were administered postoperatively. Fellow eyes were operated on during the first postoperative week using the same protocol.

Optimal IOL refractive power was determined based on thorough preoperative measurements. Data were collected from autorefractometry, pachymetry (Topcon TRK-1P, Topcon Europe Medical BV, Capelle aan den Ijssel, the Netherlands), and biometry (IOLMaster 700, Carl Zeiss Meditec AG, Jena, Germany), including axial length (AL), anterior chamber depth (ACD) keratometry values and axes (K1, K2), and white-to white distance (WTW). Anterior segment biomicroscopy and fundoscopy were performed using an Oculus Pentacam 70700 (OCULUS Optikgeräte GmbH, Wetzlar, Germany). The online Medicontur IOL Optimizer was used for Liberty IOL power calculations. We applied the Haigis formula, taking the true net power (keratometry from IOLMaster, compared with keratometries from Pentacam) into account, i.e., considering the posterior surface of the cornea. Each eye had a refractive target of emmetropia, and a surgically induced astigmatism (SIA) of +0.25 diopters (D) was included in the calculations. In cases where the calculator recommended a toric model, the Liberty 677MTY toric presbyopia-correcting IOL was chosen for implantation. Power calculations for the AT LISA tri were performed with similar parameters and using the recommendations of the IOLMaster. The toric version of the AT LISA tri (939MP) was implanted when an astigmatism-correcting IOL was recommended.

Follow-up visits were scheduled for 1 day, 1 week, and 1, 3, 6 and 12 months after surgery. The examined parameters, which were identical to those performed preoperatively, included visual acuity (uncorrected and corrected distance, intermediate, near) at 4 meters, 60 cm and 35 cm, respectively, and subjective and refractive errors (sphere, cylindrical error, spherical equivalent), also measured with an autorefractometer (Topcon TRK-1P, Topcon Europe Medical BV, Capelle aan den Ijssel, the Netherlands). Defocus curves were plotted binocularly in photopic conditions at the 12 -month visit. Contrast sensitivity measurements were performed in photopic, mesopic, and photopic conditions with glare (CSV-1000, Vector Vision Inc, Greenville, OH, USA) at 12 months following surgery. Measurements were taken at spatial frequencies of 3, 6, 12, and 18 cycles per degree (cpd) in each case (only 3, 6 and 12 cpd were measured in mesopic conditions). Optical quality, including the objective scatter index (OSI), modulation transfer function (MTF) cut-off values, and tear film analysis with tear-film OSI, were also assessed using the HD Analyzer (Visiometrics SL; Barcelona, Spain).

Detailed slit-lamp, biomicroscopy, and fundoscopy examinations of the anterior and posterior segments were carried out to identify any complications or unexpected ocular conditions. The presence of PCO was documented at each visit.

All data were collected in a Microsoft Excel file (Microsoft Inc, Redmond, WA, USA), and further statistical analysis was performed using the GraphPad Prism 9.0.1 statistical software (GraphPad Software Inc., San Diego, CA, USA). All pre- and postoperative data collected during the current study are available after de-identification in the Mendeley Data depository database from doi:10.17632/y6n5k7s6f7.1 [19].

Descriptive statistics (mean, standard deviation, median, minimum, maximum, and 95% confidence intervals) were calculated in all cases. All variables were tested for normal distribution using the D’Agostino and Pearson tests. Depending on the results, comparisons between matching pre- and postoperative variables were performed using either the paired two-tailed *t*-test (in the case of normal distribution) or the Mann–Whitney test (when a non-parametric test was required). Comparison of the two cohorts represented by the implanted IOL model was carried out using the unpaired *t*-test, the Mann–Whitney test, the Kolmogorov–Smirnov test, or the multiple unpaired *t*-test (Holm–Sidak method), based upon specific request. The visual and refractive outcomes are presented according to the standards for reporting refractive outcomes of IOL-based refractive surgery, as recently published by Reinstein et al. [20].

The results are presented as mean ± standard deviation (SD) in the case of each analysis; *p*-values of 0.05 or less were considered to be statistically significant in all cases.

## 3. Results

The Liberty IOL was implanted in 24 eyes of 12 patients, and the AT LISA tri IOL was implanted in 32 eyes (16 patients). Data were analyzed for all eyes in the AT LISA tri group and from 18 eyes of 9 patients in the Liberty group, after excluding 3 patients who were lost to follow-up after the 6-month visit. Eleven eyes of seven patients in the Liberty group, and four eyes of two patients in the AT LISA tri group required a toric IOL. As all preoperative characteristics (apart from corneal astigmatism) and postoperative results were comparable in the non-toric and toric cohorts within each IOL group, data were pooled for the analyses in each group.

Supplementary Table S2 presents the demographic data and preoperative characteristics of the two IOL groups. Average age (Liberty 56.0 ± 6.81 years; AT LISA tri 51.0 ± 7.28 years), female/male ratio (3:1), and all biometric parameters were comparable between the two groups. Preoperative uncorrected distance, uncorrected near and corrected near visual acuity (UDVA, UNVA, and CNVA) were also similar in the two groups. There was a small, but statistically significant difference in corrected distance VA (CDVA) between the Liberty and AT LISA tri groups (0.02 ± 0.05 logMAR vs. 0.11 ± 0.22; *p* = 0.0226).

### 3.1. Refractive Correction

Spherical and cylindrical errors were effectively corrected in both IOL groups. The refractive outcomes achieved at postoperative month 3 were stable through month 12 in both cohorts (Table 1). The mean spherical equivalent refraction (SEQ) was significantly closer to the target (emmetropia) in the Liberty group at both 3 and 12 months.

**Figure 1:**
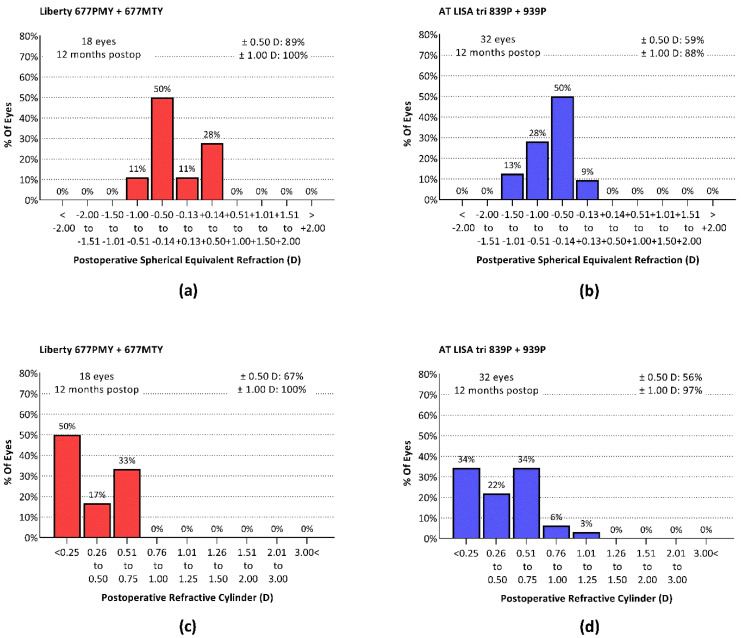
Postoperative spherical equivalent refractions and residual cylindrical refractions measured 12 months postoperatively. (**a**) Postoperative SEQ Liberty group; (**b**) postoperative SEQ AT LISA tri group; (**c**) residual astigmatism Liberty group; (**d**) residual astigmatism AT LISA tri group.

At postoperative month 12 in the Liberty group, the residual SEQ was ± 0.5 D of emmetropia in 89% of eyes and ± 1.0 D in 100% of eyes (Figure 1a). In the AT LISA tri group, residual SEQ was ± 0.5 D of emmetropia in 59% of eyes and ± 1.0 D in 88% (Figure 1b). The analysis of residual astigmatism similarly showed better outcomes in the Liberty IOL group (Figure 1c,d).

### 3.2. Visual Outcomes

Visual acuity outcomes are summarized in Table 2. UDVA and CDVA were significantly better in the Liberty group at postoperative month 3, but they did not differ between groups at month 12. Intermediate and near visual acuities were comparable in the two cohorts at both 3 and 12 months. The visual acuities at all three examined distances were stable during the follow-up period in both IOL groups.

**Figure 2:**
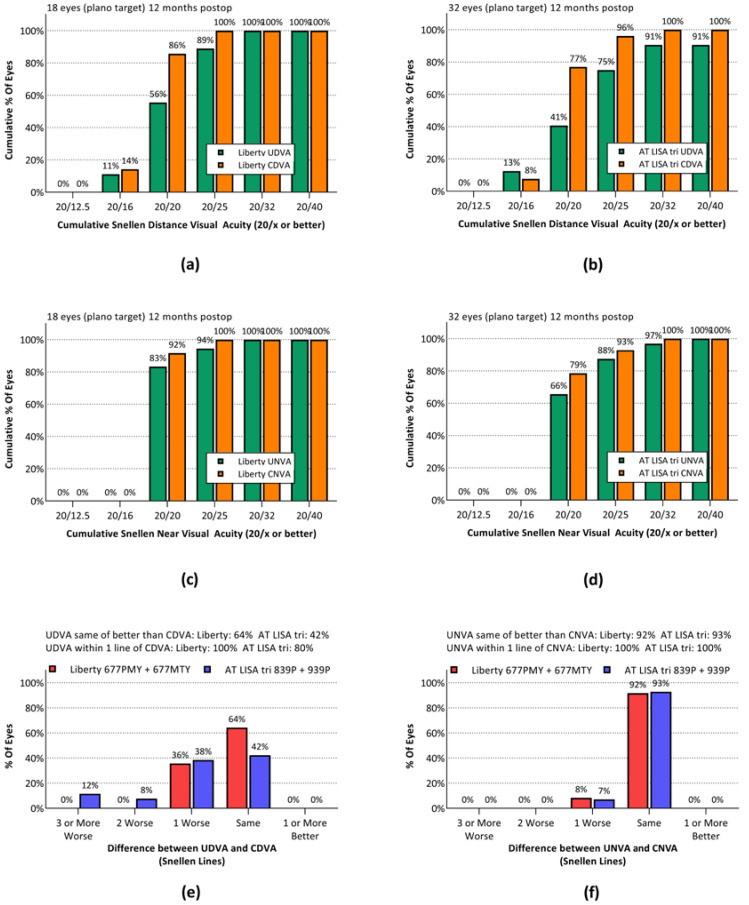
Cumulative Snellen uncorrected and corrected visual acuities for distance and near vision, measured 12 months after surgery. (**a**) Cumulative distance visual acuities for the Liberty group. (**b**) Cumulative distance visual acuities for the AT LISA tri group. (**c**) Cumulative near visual acuities for the Liberty group. (**d**) Cumulative near visual acuities for the AT LISA tri group. (**e**) Difference between UDVA and CDVA in Snellen lines for the Liberty group. (**f**) Difference between UDVA and CDVA in Snellen lines for the AT LISA tri group. UDVA: uncorrected distance visual acuity. CDVA: corrected distance visual acuity. UNVA: uncorrected near visual acuity. CNVA: corrected near visual acuity.

Snellen UDVA 20/25 or better (logMAR 0.1) was achieved by 89% of Liberty eyes and 75% of AT LISA tri eyes (Figure 2a,b). Snellen UNVA of 20/25 or better was achieved by 94% of Liberty eyes and 88% of AT LISA tri eyes (Figure 2c,d). The UDVA was within one line of the CDVA in 100% of Liberty eyes and 80% of AT LISA tri eyes (Figure 2e). In both groups, UNVA was within one line of CDVA in all eyes (Figure 2f). None of the above differences between groups achieved statistical significance.

### 3.3. Defocus Curves and Area under Curve

**Figure 3:**
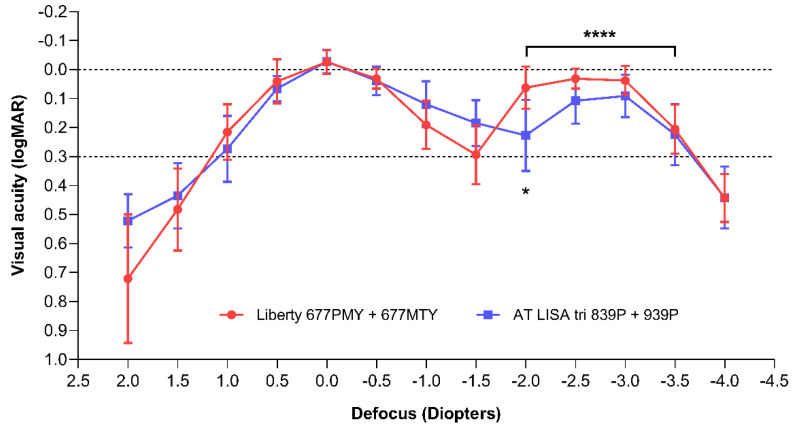
Monocular defocus curves of the two IOL models, plotted 12 months after surgery. Pairwise comparisons showed a significant difference only at the −2.0 D defocus value (*), while the area under curve analysis confirmed superior near vision performance for the Liberty IOL (*p* = 0.0004; ****).

Monocular defocus curves were plotted at 12 months postoperatively in photopic conditions (Figure 3). Multiple *t*-tests performed between the two study groups revealed a significant difference only at the −2.0 D defocus value (*p* = 0.0193). The area under the curve comparisons with a baseline of 0.3 logMAR visual acuity showed similar results in the two groups for the total (TAUC; Liberty = 1.90 ± 0.08; AT LISA tri = 1.81 ± 0.09; *p* = 0.0692), far (FAUC; Liberty = 0.68 ± 0.04; AT LISA tri = 0.65 ± 0.04; *p* = 0.1446) and intermediate range (IAUC; Liberty = 0.49 ± 0.05; AT LISA tri = 0.53 ± 0.05; *p* = 0.1446). In the near vision range (from −2.0 D to −3.5 D defocus), the Liberty IOL was associated with better results than the AT LISA tri IOL (NAUC = 0.65 ± 0.04 vs. 0.54 ± 0.06; *p* = 0.0004).

### 3.4. Visual Quality

A detailed visual quality assessment was performed at 12 months postoperatively. Contrast sensitivity was measured, and an optical quality assessment was performed. Visual functions and dysphotopsia could be evaluated based on the responses provided by 9 patients implanted with the Liberty IOLs, and 14 patients implanted with the AT LISA tri IOLs.

#### 3.4.1. Optical Quality

Detailed numerical results of the OQAS analysis are shown in Supplementary Table S3. All examined parameters (MTF cut-off, Strehl ratio, OSI, tear-film OSI, tear-film standard deviation, and tear-film difference) were comparable between the two IOL groups (*p* > 0.05).

#### 3.4.2. Contrast Sensitivity

**Figure 4:**
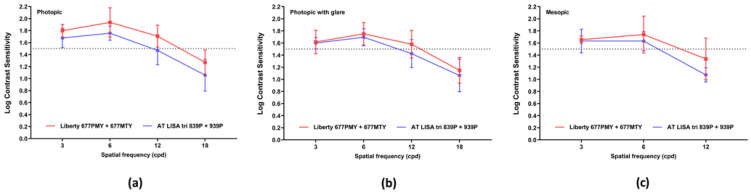
Contrast sensitivity, measured in different light conditions at 12 months postoperatively. (**a**) In photopic conditions. (**b**) In photopic conditions with glare. (**c**) In mesopic conditions (measurements not obtained at 18 cpd). cpd = cycles per degree.

Photopic, photopic with glare, and mesopic contrast sensitivities are shown in Figure 4. Contrast sensitivity was within the normal range at all measured spatial frequencies with both IOLs. Contrast sensitivity was significantly better with the Liberty than with the AT LISA tri in photopic light conditions at four out of five spatial frequencies (3 cpd: *p* = 0.0022; 6 cpd: *p* < 0.0001; 12 cpd: *p* = 0.0019; 18 cpd: *p* = 0.0060).

#### 3.4.3. Visual Function, Dysphotopsia, Patient Satisfaction

Patient self-assessments of postoperative visual function and dysphotopic events were collected using a modified visual function questionnaire completed at postoperative month 12. Each item and the possible responses are listed in Supplementary Table S4. Responses were evaluable for 9 patients in the Liberty group and 14 patients in the AT LISA tri group.

**Figure 5:**
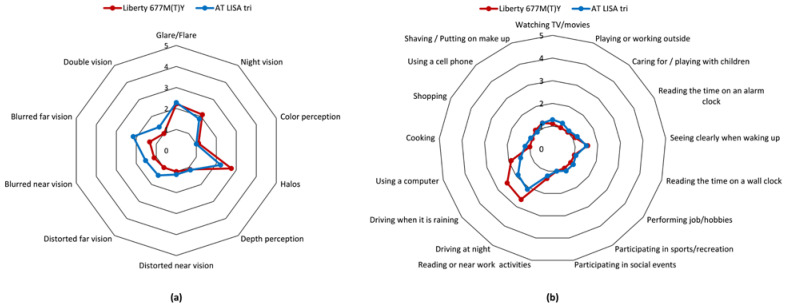
Mean responses for (**a**) dysphotopsia and (**b**) visual function assessment using a modified visual function questionnaire completed at 12 months postoperatively. 1 = No difficulties; 2 = minor difficulties; 3 = moderate difficulties; 4 = major difficulties; 5 = cannot accomplish task. No significant differences between the two lenses could be revealed.

Dysphotopic events and other visual disturbances, such as blurred or distorted vision, were rarely reported with either IOL (Figure 5a), and there were no significant differences between the responses given by the patients in the two study groups (Supplementary Table S5). The majority of daily tasks could be performed with no difficulty to only minor difficulties by the vast majority of patients in both IOL groups (Figure 5b).

**Figure 6:**
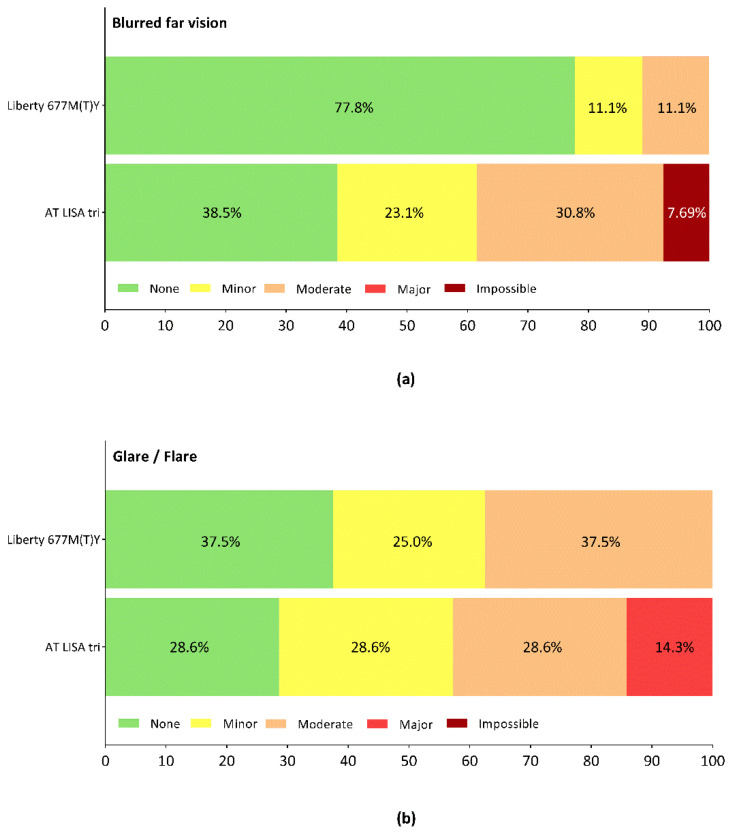
Distribution of responses given by the patients regarding difficulties caused by (**a**) blurred far vision, and (**b**) glare/flare. Subjective assessment was performed using a modified visual function questionnaire completed at 12 months postoperatively.

Blurred far vision was reported by a minority of the Liberty patients, and more often by patients in the AT LISA tri group (Figure 6a; Supplementary Table S5). Glare and flare were the most common dysphotopic phenomena, although most patients rated them as representing no, minor, or moderate difficulties. Major concerns were only reported by AT LISA tri patients (Figure 6b).

**Figure 7:**
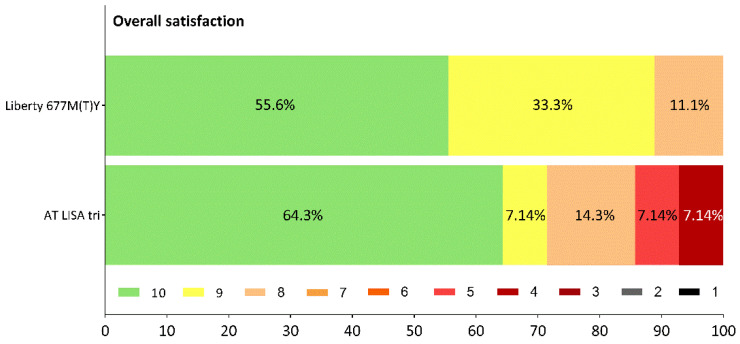
Overall satisfaction reported by patients at 12 months after surgery. 10 = highest ranking; 1 = lowest ranking.

The majority of patients were highly satisfied with the surgical outcomes and their postoperative quality of vision (Figure 7). Only a few complaints came from the AT LISA tri cohort: although all patients (100%) from the Liberty group achieved spectacle independence for far and near vision, and only one patient of the nine (11.1%) required spectacles for intermediate vision from time to time, 14.3% of the AT LISA tri subjects often needed their glasses for far vision and 7.1–7.1% often needed to wear spectacles for intermediate and near vision.

### 3.5. Posterior Capsule Opacification

During the first 12 months after surgery, PCO developed in 6 (33.3%) of 18 eyes implanted with the Liberty IOL and 20 (62.5%) of 32 eyes in the AT LISA tri group. The percentage of eyes that had Nd:YAG capsulotomy for visually significant PCO was significantly lower in the Liberty group than in the AT Lisa tri group (16.7% vs. 40.7%; *p* = 0.1171) The mean time to development of PCO elapsing between the cataract surgery and PCO development was 10.1 ± 1.41 months in the Liberty, group and 8.61 ± 4.02 months in the AT LISA tri group cohort; however, this difference was not significant (*p* = 0.8000).

No serious complications related to either the surgery or the IOLs occurred. Across both IOL groups, corneal edema and increased IOP were seen in early follow-up in five and four eyes, respectively, with resolution by the 1-month postoperative visit. Dry eye syndrome was diagnosed at the 12-month visit in four eyes of three patients in the Liberty group and in six eyes of three patients in the AT LISA tri group.

## 4. Discussion

Our study aimed to characterize and compare the clinical outcomes and quality of vision after the implantation of one of two trifocal IOLs. It includes data from 50 eyes of 25 patients followed up to 12 months after surgery.

Both investigated IOLs effectively corrected refractive and cylindrical errors. However, the Liberty IOL was associated with better refractive predictability. Our results show that the residual SEQ refraction was within 0.5 D of the target in 89% of Liberty eyes and 59% of AT LISA tri eyes. These results are not as good as those reported in a study by Serdiuk et al., where 97% of Liberty eyes and 71% of AT LISA tri eyes achieved a residual SEQ ≤ 0.5 D [21]. Nevertheless, a difference favoring the Liberty IOL is apparent in both studies [21]. Astigmatism outcomes in our study also showed differences between the two IOL models, with better results achieved with the Liberty IOL These results suggest that the Liberty lens is associated with more predictable refractive outcomes and a lower risk of postoperative refractive surprises. However, further studies are needed to confirm these findings. Refractive outcomes achieved by the end of the third postoperative month were shown to be maintained with both examined lenses during the entire follow-up period.

Visual outcomes reflect the refractive results. Both IOLs provided sharp vision at multiple distances (far, intermediate, near), and the uncorrected distance, intermediate and near visual acuities are in good agreement with previously published data for these lenses [21,22,23,24,25,26,27,28]. Nevertheless, a remarkably higher proportion of eyes achieved good functional monocular vision at multiple distances with the Liberty lens compared to the plate-haptic AT LISA tri. At 12 months following implantation, 56% of Liberty eyes and 41% of AT LISA tri eyes had a monocular distance vision of at least 20/20 (Snellen), and 89% of Liberty eyes vs. 75% of AT LISA tri eyes achieved at least 20/25. These results are inferior to those reported by both Serdiuk et al. and Piovella et al. [21,22]. However, the latter paper reported binocular results, which are usually more favorable than monocular visual acuities [22].

The intermediate vision results have to be interpreted with consideration of the testing distance. Some studies report data with intermediate vision measured at 80 cm, while others publish data measured at 66 cm from the eye [21,22,28,29,30]. As the AT LISA tri lens has a +1.66 D addition and the Liberty IOL has a +1.75 D addition calculated at the IOL plane, we find it more expedient to focus on the intermediate vision data measured at 66 cm, which is roughly equivalent to a +1.50 D addition on the defocus curve. Liberty eyes have given somewhat lower results with the +1.50 D addition compared to the AT LISA tri IOL; however, we could not find a significant difference. We did find that patients implanted with the Liberty IOL had significantly better visual performance in the near vision range compared to the AT LISA tri group in 83% of Liberty eyes, but only 66% of AT LISA tri eyes achieved monocular UDVA of 20/20 or better, while 94% of Liberty eyes and 88% of AT LISA tri eyes achieved UNVA of 20/25 or better. Our results are superior to those reported by other authors, although any attempt to make comparisons is limited by a lack of detail regarding their exact measurement conditions and patient population characteristics [21,22].

The results of defocus curve testing are consistent with the VA measurements; the results for VA show that both IOLs provide excellent distance vision. Performance in the intermediate range seems somewhat inferior for the Liberty IOL compared to the AT LISA tri, but the difference is not statistically significant. Furthermore, the visual function assessment results of the two examined lenses are also comparable—not only in the intermediate range but also for near and far distances. The near vision range provided by the Liberty IOL in our study (from about −1.75 D to −3.5 D defocus) was wider than that of the AT LISA tri and similar to that previously reported by Fernández et al. [24]. This difference may explain the higher percentage of Liberty patients achieving spectacle independence for near vision compared to the AT LISA tri group (100% vs. 92.9%). In addition, freedom from glasses for far vision was reported by all patients in the Liberty group, whereas14.3% of the AT LISA tri patients often required additional correction for far vision.

An optical quality assessment performed using an OQAS system gave identical results for the two lenses, which are made from the same material. Nevertheless, differences between the two IOL groups in visual quality characterized by subjective parameters, such as contrast sensitivity and visual disturbances were recorded. We postulate the differences may be explained by the potential for IOL design and optical surface features to impact visual performance. Mesopic and photopic glare contrast sensitivities were in the physiological range and were similar for both lenses. The photopic contrast sensitivity values, however, were better with the Liberty lens. The latter result is in agreement with the findings of other investigations, including studies reporting lower contrast sensitivity values for the AT LISA tri than we found and a reduction at high spatial frequencies (12 and 18 cpd) [22,26,28]. The contrast sensitivity curves for the Liberty IOL in our patients match those presented in other studies of this lens [23,25].

It is well known that multifocal IOLs are frequently associated with photic disturbances, and that the frequency and intensity of these visual symptoms have a high impact on overall visual quality and hence on patient satisfaction [31,32,33,34,35,36,37,38]. Although the results for dysphotopsia and visual functions were similar for the Liberty and AT LISA tri IOLs, we recognized that patient responses usually lack extreme ratings in the case of the Liberty IOL. We speculate that better refractive predictability with the Liberty IOL might account for this difference. Although further investigation is required to gather evidence that would support this explanation, patients with higher residual refractive error are more prone to report dysphotopsia and be less satisfied with their overall visual quality [37,38].

The explanation for the better refractive predictability of the Liberty IOL is also unclear. Comparing two MFIOLs, Meng et al. reported that a plate haptic lens had better positional stability in the capsular bag than a lens with a C-loop haptic in myopic eyes (AXL > 24.5 mm) and noted no differences in non-myopic eyes (21.0 mm < AXL ≤ 24.5 mm) [39]. Because AXL was similar in the two IOL groups in our study (*p* = 0.6400), we believe that the differences between groups in the refractive and visual outcomes are not attributable to this anatomical parameter. IOL decentration and tilt were not measured in our study, although both the Liberty and AT LISA tri IOLs have been reported to undergo some decentration following implantation [24,39]. IOL decentration can affect refractive and visual outcomes [38,40]. Kim et al. reported a tendency towards myopia among patients implanted with the AT LISA tri 839MP compared to a group implanted with a C-loop haptic IOL [26].

IOL design is also known to affect PCO development [41,42,43,44,45]. In our cohort, one-third of the Liberty eyes developed PCO during the first postoperative year, and half of these eyes required surgical treatment, namely, Nd:YAG laser capsulotomy. In contrast, almost two-thirds of the AT LISA tri eyes developed PCO, and two-thirds of these eyes had a perceptible decrease in vision that required surgical management. Because all surgeries were performed by the same experienced surgeon using the same surgical protocol with the utmost caution, we believe that the differences in frequency and severity of PCO are likely explained by differences in IOL design [16,46]. We acknowledge that our reported 1-year rates for PCO and Nd:YAG capsulotomy are higher than those reported in other studies [29,47,48,49]. The definition of PCO and the criteria for performing Nd:YAG capsulotomy, however, may differ between centers.

Our study has its limitations. Patients could not be randomized to an IOL group. However, all surgeries and examinations were performed by the same personnel using the same protocol, equipment, and tools, in order to minimize variations that might affect outcomes. Moreover, our study includes a relatively small number of patients, and therefore our findings should be interpreted carefully. Further investigations including more patients should be performed to confirm the current results. Nevertheless, our clinical investigation provides a detailed and comprehensive comparison of outcomes achieved with the two examined trifocal IOLs.

Our results indicate that both investigated IOLs offer safe and effective presbyopia correction after cataract surgery. Because the IOLs are made of the same material, they seem to provide comparable optical quality, and the majority of patients are highly satisfied with their visual function. Differences in visual acuities at different ranges and contrast sensitivities favoring the Liberty group were observed and may be explained by the different optical surfaces of the two presbyopia-correcting IOL models. Differences in haptic design may explain the remarkable differences between groups in PCO development and severity: the double-loop haptic IOL seems to provide more efficient PCO prevention than the IOL with plate haptics.

## 5. Conclusions

Although several presbyopia-correcting IOLs are available on the market, and most of them are able to provide good functional vision at multiple distances, there may be differences in the quality of vision they produce. The visual outcomes seem to reflect differences in the optical technology and other design elements implemented by the manufacturer. Surgeons should be aware of possible differences between trifocal IOLs with respect to clinical outcomes, including the possible side effects of dysphotopsia and reduced contrast sensitivity, and choose the optimal model based on the unique ocular characteristics and visual preferences of each patient.

## Figures and Tables

**Table 1:** Postoperative residual spherical (SPH), cylindrical (Cyl) and spherical equivalent (SEQ) refractions measured 3 and 12 months postoperatively. Data are presented as mean ± SD.

Residual Refraction	IOL	Month 3	Month 12	Month 3 vs. Month 12 *p* = ^1^
SPH	Liberty	0.05 ± 0.21	−0.47 ± 0.47	0.6193
	AT LISA tri	−0.06 ± 0.36	−0.27 ± 0.43	0.3189
	*p* = ^2^	0.0004 *	0.0739	
Cyl	Liberty	−0.39 ± 0.63	−0.44 ± 0.27	0.3276
	AT LISA tri	−0.35 ± 0.37	−0.54 ± 0.30	0.0879
	*p*=	0.7865	0.0499 *	
SEQ	Liberty	−0.20 ± 0.32	−0.23 ± 0.39	>0.9999
	AT LISA tri	−0.24 ± 0.39	−0.60 ± 0.44	0.6062
	*p*=	0.0021 *	0.0054 *	

^1^ Comparison of the residual refractions measured during the two visits was performed using the paired two-tailed Wilcoxon or Mann–Whitney test. *p*-values of ≤ 0.05 were considered as statistically significant (*). ^2^ Comparison of the residual refractions measured in the two IOL-groups was performed using the Kolmogorov–Smirnov test. *p*-values of ≤ 0.05 were considered as statistically significant (*).

**Table 2:** Monocular distance (logMAR) and near (Jaeger) visual acuities, measured 3 and 12 months postoperatively. Data are presented as mean ± SD.

**Visual Acuity**	**IOL**	**Month 3**	**Month 12**	**Month 3 vs. Month 12 *p* = ^1^**
UDVA	Liberty	0.02 ± 0.04	0.03 ± 0.07	0.0469 *
	AT LISA tri	0.13 ± 0.06	0.10 ± 0.19	0.3125
	*p* = ^2^	0.0033 *	0.2206	
CDVA	Liberty	0.00 ± 0.03	0.00 ± 0.04	>0.9999
	AT LISA tri	0.04 ± 0.06	0.01 ± 0.05	0.2500
	*p* = ^2^	0.0394 *	0.4720	
DCIVA	Liberty	N/A	0.30 ± 0.10	N/A
	AT LISA tri	N/A	0.18 ± 0.08	N/A
	*p* = ^2^	N/A	0.0001 *	
	Liberty	0.02 ± 0.05	0.02 ± 0.05	>0.9999
UNVA	AT LISA tri	0.00 ± 0.00	0.05 ± 0.08	0.2500
	*p* = ^2^	0.2258	0.2092	
	Liberty	0.03 ± 0.06	0.01 ± 0.03	0.3714
CNVA	AT LISA tri	0.00 ± 0.00	0.03 ± 0.06	0.5539
	*p* = ^2^	0.4286	0.5252	

^1^ Comparison of the visual acuities measured during the two visits was performed using the paired two-tailed Wilcoxon or Mann–Whitney test. *p*-values of ≤ 0.05 were considered statistically significant (*). ^2^ Comparison of the visual acuities measured in the two IOL-groups was performed using the Kolmogorov–Smirnov test. *p*-values of ≤ 0.05 were considered statistically significant (*). UDVA: uncorrected distance visual acuity. CDVA: corrected distance visual acuity. DCIVA: distance-corrected intermediate visual acuity. UNVA: uncorrected near visual acuity. CNVA: corrected near visual acuity. N/A: not applied.

## Data Availability

Data are available in a publicly accessible repository. The data presented in this study are openly available on Mendeley Data at doi:10.17632/y6n5k7s6f7.1.

## Supplementary Materials

The following are available online at https://www.mdpi.com/article/10.3390/jcm10153268/s1, Table S1: Technical data of the two investigational devices, Table S2: Demographic and preoperative characteristics of the study population, Table S3: Optical quality assessment 12 months following IOL-implantation has shown that all examined parameters are comparable between the two IOL-subsets of patients, Table S4: Visual function self-assessment questionnaire completed by the patients 12 months following IOL implantation, Table S5: Visual function self-assessment of the patients 12 months following IOL implantation.
