# Supplementary material for: Comparison of Clinical Outcomes, Visual Quality and Visual Function of Two Presbyopia-Correcting Intraocular Lenses Made from the Same Material, but with Different Design and Optics
Source: J Clin Med. 2021 Jul 24;10(15):3268. doi: 10.3390/jcm10153268 (PMC8347739; doi:10.3390/jcm10153268)
Supplement: Supplementary file 1 [file jcm-10-03268-s001.zip › jcm-1264214-supplementary.pdf]

Supplementary Table S1. Technical data of the two investigational devices.

	**Liberty 677PMY/677MTY**	**AT LISA tri 839MP/939MP**
Diffractive technology	Diffractive trifocal up to 3.0 mm, thereafter monofocal	Diffractive trifocal up to 4.34 mm, thereafter bifocal
Diffractive steps	7	29
Diffractive orders	2 (order 0 and 1)	3 (order 0, 1 and 2)
Diffractive surface	anterior surface, Ø3.0 mm	anterior surface, Ø4.34 mm
Light loss (3.0 mm pupil)	11%	14.3%
Light energy split (3.0 mm pupil)	53%D/14%I/33%N	50%D/20%I/30%N
Optic add powers (D)	+1.75 D and +3.50 D	+1.66 D and +3.33 D
Toric available	Yes	Yes
Reading distance (cm)	37.5 cm	40 cm
Aberration correction	Aberration neutral	-0.20 µm
Lens material	Hydrophilic Acrylic (Benz-25)	Hydrophilic Acrylic (Benz-25)
Abbe-number	58	58
Refractive index	1.46	1.46
Filtration	UV and blue light (390-460 nm)	UV
Dioptre range	0.0 D - +35.0 D	0.0 D - +32.0 D
Optic/Haptic diameter	6.00 mm /13.0 mm	6.00 mm /11.0 mm
Haptic design	Specific double loop	Plate
Injection	Preloaded	Preloaded

Supplementary Table S2. Demographic and preoperative characteristics of the study population.

Demographic	Liberty 677PMY/677MTY		AT LISA tri 839MP/939MP		p=[7]
	**Mean ± SD**	**Range**	**Mean ± SD**	**Range**	
Age (years)	56.0 ± 6.81	47 - 69	51.0 ± 7.28	52 - 75	0.0761
Female/Male	9 females (75%)<br>3 males (25%)		12 females (75%)<br>4 males (25%)		>0.9999
Axial length (AXL; mm)	23.05 ± 0.75	21.99 – 24.46	23.14 ± 0.63	22.28 – 24.29	0.6400
Anterior chamber depth (ACD)	2.95 ± 0.22	2.57 – 3.24	3.10 ± 0.41	2.37 – 4.23	0.1130
K1 (mm)[1]	7.89 ± 0.27	7.45 – 8.52	7.88 ± 0.23	7.42 – 8.37	0.7958
K2 (mm)[1]	7.75 ± 0.29	7.26 – 8.45	7.77 ± 0.22	7.32 – 8.30	0.5903
Corneal thickness (µm)	533.5 ± 19.67	508 - 569	565.4 ± 28.33	513 - 627	0.6844
Intraocular pressure (IOP; mmHg)	16.67 ± 2.60	13 - 23	16.03 ± 2.75	10 - 21	0.3853
Pupil size photopic (mm)	3.31 ± 1.50	2.28 – 7.70	2.71 ± 0.48	2.06 – 3.53	0.1749
Pupil size mesopic (mm)	5.06 ± 1.20	4.10 – 8.20	N/A	N/A	N/A
Spherical error (SPH; D)	1.94 ± 0.96	0.00 – +4.00	1.89 ± 1.68	-1.25 – +7.00	0.6148
Cylindric error (Cyl; D)	-0.49 ± 0.22	-1.00 - -0.25	-0.45 ± 0.48	-1.50 - +0.50	0.7696
Spherical equivalent (SEQ; D)	1.79 ± 0.95	-0.25 - +3.75	1.78 ± 01.77	-1.75 - +7.00	0.7041
UDVA (logMAR)[2]	0.32 ± 0.17	0.00 – 0.52	0.38 ± 0.27	0.00 – 1.00	0.8137
CDVA (logMAR)[3]	0.02 ± 0.05	0.00 – 0.22	0.11 ± 0.22	0.00 – 1.00	0.0226*
UNVA (Jaeger)[4]	8.50 ± 2.52	6 - 12	N/A	N/A	N/A
CNVA (Jaeger)[5]	1.41 ± 0.73	1 - 3	1.27 ± 0.63	1 - 3	0.6053
IOL power (SEQ; D)[6]	23.1 ± 2.01	19.0 – 26.5	22.9 ± 2.47	19.5 – 29.0	0.8173
IOL cylinder (Cyl; D)	0.54 ± 0.62	0.00 – 1.50	0.14 ± 0.39	0.00 – 1.50	0.0049*

[1] Keratometry values
[2] Uncorrected Distance Visual Acuity
[3] Corrected Distance Visual Acuity
[4] Uncorrected Near Visual Acuity
[5] Corrected Near Visual Acuity
[6] Intraocular Lens
[7] p-values of the unpaired t-tests or Mann-Whitney test between the two study groups.
P-values of ≤0.05 were considered as statistically significant (*).

Supplementary Table S3. Optical quality assessment 12 months following IOL-implantation has shown that all examined parameters are comparable between the two IOL-subset of patients.

Optical quality parametres	Liberty 677PMY/677MTY				AT LISA tri 839MP/939MP				p=[3]
	**Mean**	**SD**	**Min**	**Max**	**Mean**	**SD**	**Min**	**Max**	
**Month 3**									
MTF cut-off	32.3	13.9	5.33	56.4	34.3	10.5	14.4	54.8	0.5324
Strehl-ratio	0.20	0.08	0.05	0.33	0.19	0.07	0.09	0.35	0.6671
OSI	2.26	1.36	0.00	5.17	1.60	0.43	0.81	2.21	0.1061
Tear-film OSI mean	2.44	0.98	0.75	3.99	1.96	0.65	0.92	2.79	0.1467
Tear-film OSI standard deviation	0.43	0.35	0.05	0.23	0.33	0.19	0.08	0.69	0.4002
Tear-film OSI difference	1.60	1.03	0.23	4.15	1.41	0.85	0.34	2.65	0.5937
**Month 12**									
MTF cut-off	34.5	12.1	9.30	54.3	31.8	11.9	12.9	56.2	0.2124
Strehl-ratio	0.20	0.07	0.06	0.35	0.18	0.08	0.09	0.36	0.1239
OSI	2.11	1.62	0.00	6.71	2.03	0.95	0.69	4.40	0.7901
Tear-film OSI mean	1.96	1.11	0.00	4.82	2.44	0.96	1.12	5.38	0.0884
Tear-film OSI standard deviation	0.54	0.74	0.00	2.92	0.40	0.28	0.10	1.36	0.3404
Tear-film OSI difference	2.79	4.71	0.34	17.7	1.62	0.96	0.45	4.92	0.1822

[1] Modular Transfer Function

[2] Objective Scatter Index

[3] p-values of the unpaired t-tests or Mann-Whitney test between the two study groups. P-values of ≤0.05 were considered as statistically significant (*).

Supplementary Table S4. Visual function self-assessment questionnaire completed by the patients 12 months following IOL-implantation.

A	Visual Task Difficulty Assessment					
How much difficulty have you got with each of the following?						
		1 No difficulty	2 Minor difficulty	3 Moderate difficulty	4 Major difficulty	5 Cannot accomplish	6 Not applicable
1.	Glare/Flare (trouble seeing street signs due to bright light or oncoming headlight)					
2.	Night vision					
3.	Recognizing specific colors (color perception)					
4.	Rings around light sources (halos)					
5.	Lining things up, pouring liquids or going down stairs (depth perception)					
6.	Straight lines look crooked close up (distorted near vision)					
7.	Straight lines look crooked at distance (distorted far vision)					
8.	Blurred near vision					
9.	Blurred far vision					
10.	Double vision					

B	Visual Lifestyle Activities						
How much difficulty have you got with each of the following activities due to your vision (without your spectacle sor contact lenses)?							
		1 No difficulty	2 Minor difficulty	3 Moderate difficulty	4 Major difficulty	5 Cannot accomplish	6 Not applicable
1.	Watching TV or movies						
2.	Playing or working outdoors						
3.	Caring for/playing with children						
4.	Reading the time on an alarm clock						
5.	Seeing clearly when waking up						
6.	Performing your job / hobbies						
7.	Participating in sports or other recreational activities						
8.	Participating in social events						
9.	Reading and near work activities						
10.	Driving at night						
11.	Driving when it is raining						
12.	Using a computer						
13.	Cooking						
14.	Shopping						
15.	Using a cell phone						
16.	Shaving / putting on make-up						

C	Spectacle use					
How often do you use spectacles or contact lenses?						
		1 Always	2 Often	3 Seldom	4 Rare	5 Never
1.	For distance vision					
2.	For intermediate vision					
3.	For near vision					

D	Overall satisfaction
How much satisfied are you with your current vision on a scale from 1 to 10 (1=at least; 10= maximum satisfaction)?	

Supplementary Table S5. Visual function self-assessment of the patients 12 months following IOL-implantation.

Visual function items	Liberty 677PMY/677MTY				AT LISA tri 839MP/939MP				p=[1]
	Mean	SD	Min	Max	Mean	SD	Min	Max	
Glare/Flare	2.22	1.09	1	4	2.29	1.07	1	4	>0.9999
Night vision	2.11	0.78	1	3	1.86	0.53	1	3	0.8466
Color perception	1.11	0.33	1	2	1.00	0.00	1	1	>0.9999
Halos	2.75	0.89	1	4	2.21	0.89	1	4	0.4118
Depth perception	1.11	0.33	1	2	1.14	0.36	1	2	>0.9999
Distorted near vision	1.00	0.00	1	1	1.14	0.36	1	2	0.9999
Distorted far vision	1.00	0.00	1	1	1.46	0.88	1	4	0.6952
Blurred near vision	1.11	0.33	1	2	1.54	0.78	1	3	0.8212
Blurred far vision	1.33	0.71	1	3	2.15	1.21	1	5	0.3836
Double vision	1.00	0.00	1	1	1.38	0.77	1	3	0.9396
Watching TV/movies	1.11	0.33	1	2	1.29	0.61	1	3	>0.9999
Playing or working outside	1.00	0.00	1	1	1.21	0.58	1	3	0.9999
Caring for / playing with children	1.00	0.00	1	1	1.07	0.27	1	2	>0.9999
Reading the time on an alarm clock	1.11	0.33	1	2	1.21	0.43	1	2	>0.9999
Seeing clearly when waking up	1.56	0.53	1	2	1.50	0.65	1	3	>0.9999
Reading the time on a wall clock	1.00	0.00	1	1	1.07	0.27	1	2	>0.9999
Performing job/hobbies	1.00	0.00	1	1	1.14	0.36	1	2	0.9999
Participating in sports/recreation	1.00	0.00	1	1	1.14	0.36	1	2	0.9999
Participating in social events	1.00	0.00	1	1	1.00	0.00	1	1	>0.9999
Reading or near work activities	1.33	0.71	1	3	1.21	0.58	1	3	>0.9999
Driving at night	2.63	0.92	1	4	2.10	0.57	1	3	0.1448
Driving when it is raining	2.50	0.76	2	4	1.90	0.74	1	3	0.2986
Using a computer	1.89	0.93	1	3	1.46	0.78	1	3	0.8995
Cooking	1.00	0.00	1	1	1.21	0.58	1	3	0.9999
Shopping	1.00	0.00	1	1	1.07	0.27	1	2	>0.9999
Using a cell phone	1.11	0.33	1	2	1.00	0.00	1	1	>0.9999
Shaving / Putting on make up	1.22	0.67	1	3	1.21	0.58	1	3	>0.9999
Spectacle use - Distance	5	0	5	5	4.86	0.363	4	5	0.9999
Spectacle use - Intermediate	4.778	0.667	3	5	4.71	1.069	1	5	>0.9999
Spectacle use - Near	5	0	5	5	4.71	1.069	1	5	>0.9999
Overall satisfaction	9.39	0.70	8	10	8.86	1.99	4	10	0.9823

[1] p-values of the unpaired t-tests or Mann-Whitney test between the two study groups. P-values of ≤0.05 were considered as statistically significant (*).
